# ISCEV Standard for full-field clinical electroretinography (2022 update)

**DOI:** 10.1007/s10633-022-09872-0

**Published:** 2022-05-05

**Authors:** Anthony G. Robson, Laura J. Frishman, John Grigg, Ruth Hamilton, Brett G. Jeffrey, Mineo Kondo, Shiying Li, Daphne L. McCulloch

**Affiliations:** 1 Department of Electrophysiology, Moorfields Eye Hospital, 162 City Road, London, EC1V2PD UK; 2 Institute of Ophthalmology, University College London, London, UK; 3 University of Houston College of Optometry, Houston, TX USA; 4 Ophthalmic Genetics and Visual Function Branch, National Eye Institute, National Institutes of Health, Bethesda, MD 20892-1860 USA; 5 Save Sight Institute Faculty of Medicine and Health, University of Sydney, Sydney, NSW Australia; 6 Department of Clinical Physics and Bioengineering, Royal Hospital for Children, NHS Greater Glasgow and Clyde, Glasgow, UK; 7 College of Medical, Veterinary and Life Sciences, University of Glasgow, Glasgow, UK; 8 Mie University Graduate School of Medicine, Tsu, Japan; 9 Department of Ophthalmology, Xiang’an Hospital of Xiamen University, Fujian, China; 10 Eye Institute of Xiamen University (EIXU), Fujian, China; 11 School of Optometry and Vision Science, University of Waterloo, Waterloo, Canada

**Keywords:** Clinical standards, Electroretinogram (ERG), Full-field ERG, International Society of Clinical Electrophysiology of Vision (ISCEV)

## Abstract

The full-field electroretinogram (ERG) is a mass electrophysiological response to diffuse flashes of light and is used widely to assess generalized retinal function. This document, from the International Society for Clinical Electrophysiology of Vision (ISCEV), presents an updated and revised ISCEV Standard for clinical ERG testing. Minimum protocols for basic ERG stimuli, recording methods and reporting are specified, to promote consistency of methods for diagnosis, monitoring and inter-laboratory comparisons, while also responding to evolving clinical practices and technology. The main changes in this updated ISCEV Standard for clinical ERGs include specifying that ERGs may meet the Standard without mydriasis, providing stimuli adequately compensate for non-dilated pupils. There is more detail about analysis of dark-adapted oscillatory potentials (OPs) and the document format has been updated and supplementary content reduced. There is a more detailed review of the origins of the major ERG components. Several tests previously tabulated as additional ERG protocols are now cited as published ISCEV extended protocols. A non-standard abbreviated ERG protocol is described, for use when patient age, compliance or other circumstances preclude ISCEV Standard ERG testing.

## Introduction

Full-field electroretinography (ERG) is an established clinical technique [1] used to evoke and record mass responses of the retina to flashes of light. The first ISCEV Standard for clinical ERG was published in 1989 to promote conformity of methods and to facilitate inter-laboratory comparisons of ERG data [2]; it was last updated in 2015 [3]. This document updates the ISCEV Standard for clinical ERG testing and supersedes the 2015 version. It defines minimum protocols for clinical ERG stimuli, recording and reporting, to allow reproducible recordings that can be recognized and compared from different laboratories worldwide. Standardization also allows direct comparisons with high quality legacy data for clinical monitoring and research purposes. These benefits must be balanced with the need to respond to changing clinical practices and advances in knowledge and technology.

In addition to the ISCEV Standard ERG, extended protocols may be indicated to enhance patient management, or for detailed characterization such as functional phenotyping or clinical trials. ISCEV extended ERG protocols currently include those for the photopic negative response [4], the dark-adapted red flash ERG [5], the photopic On–Off ERG [6], stimulus–response series for light-adapted full-field ERG [7] and for the dark-adapted full-field ERG b-wave [8], the S-cone ERG [9], and for the derivation and analysis of the strong flash rod-isolated ERG a-wave [10].

ISCEV publishes and maintains other standards for clinical electrophysiological tests, often used to complement full-field ERG testing. These include standards for pattern electroretinography [11], multifocal electroretinography [12], electro-oculography [13] and visual evoked potentials (VEP) [14]. There is also an extended protocol for VEP methods of estimation of visual acuity [15] and a technical and calibration guideline for use in clinical electrophysiology [16]. The ISCEV guide to electrodiagnostic procedures highlights the typical clinical applications of all ISCEV Standard clinical tests including the full-field ERG [1]. The ISCEV website should be consulted for current updates (www.ISCEV.org/standards). This document is not a safety standard, and it does not mandate procedures for individual patients or define the qualifications required of those administering or interpreting the tests.

## Summary of changes to the ISCEV Standard full-field ERG

The main revisions in this Standard include accepting an alternative to the requirement for mydriasis to control retinal illumination and more detail about analysis of dark-adapted oscillatory potentials (OPs). The document format has been updated and supplementary content removed and largely incorporated within the main text. This revision contains more detail about the origins of ERGs as well as updates to the figure, table and references. A non-standard abbreviated ERG protocol is described for use when the ISCEV Standard method is not possible, e.g. when precluded by a patient’s young age or inability to comply with the Standard protocols.

## Overview and retinal origins of the Standard full-field ERGs

The ISCEV Standard full-field ERGs assess generalized retinal function under dark-adapted (DA) and light-adapted (LA) conditions. A ganzfeld (whole field) stimulator delivers diffuse flashes that evenly illuminate the maximal area of retina. Responses to the flashes are recorded with electrodes in contact with the cornea or bulbar conjunctiva or with skin electrodes attached to the lower eyelids. The ISCEV Standard protocol includes a minimum period of 20 min dark adaptation to enable assessment of DA (rod-dominated) retinal function, either before or after LA testing. If recorded after the DA ERGs, a minimum period of 10 min light adaptation minimizes rod contributions and optimizes the selective contributions of the LA cone system. The DA ERGs include responses to flash strengths (in photopic units; phot) of 0.01, 3 and 10 phot cd·s·m^-2^ (DA 0.01; DA 3; DA 10). The LA ERGs are to a flash strength of 3 phot cd·s·m^-2^, superimposed on a light-adapting background (luminance 30 cd·m^-2^) as single flashes (LA 3 ERG) and at a frequency close to 30 Hz (LA 30 Hz ERG).

After the period of dark adaptation, a dim flash, below the threshold for the DA cone system, is used to elicit the DA 0.01 ERG, arising in the rod bipolar cells but dependent on input from functional rod photoreceptors. The DA 3 and DA 10 ERGs are mixed rod and cone system responses but in a healthy retina the rod system contribution dominates. Both have cornea-negative a-waves but the DA 10 ERG a-wave is larger and of shorter peak time, consistent with a greater rod photoreceptor contribution. Both have cornea-positive b-waves that arise largely in the rod-driven On-bipolar cells. The DA 10 ERG may be more informative than ERGs to weaker flashes in patients with opaque media, small pupils or immature retinae. Within the ERG waveform there are relatively high frequency and relatively low amplitude components on the rising limb of the DA 3 and DA 10 ERG b-waves, named oscillatory potentials. The cellular origins of the OPs have yet to be fully established but appear to reflect inner retinal activity involving amacrine cells and retinal ganglion cells. For the stronger flash (DA 10), the OPs are typically larger, and the b-wave to a-wave amplitude ratio smaller than for the DA 3 stimulus.

Light-adapted testing includes two types of ERG recorded to provide different but complementary measures of cone system function. A flash presented close to 30 Hz, superimposed on a light-adapting background, is used to elicit the LA 30 Hz ERG, generated largely by cone On- and Off- bipolar cells but dependent on the function of the long- and medium- wavelength sensitive cones (L- and M- cones). The short-wavelength sensitive cone (S-cone) system has lower temporal resolution and makes a minimal contribution to the LA 30 Hz ERG. The single flash LA 3 ERG has an a- and b- wave. The LA 3 ERG a-wave is dominated by the activity of the Off- bipolar cells; the b-wave is dominated by a combination of On- and Off- bipolar cell activity, with contributions mediated by L-, M- and S-cone mechanisms. Full-field ERGs are generated across the entire retina with minimal contribution from the macula (although cone density is greatest at the fovea, a large majority of cones are located outside the central retina). Electrophysiological assessment of central retinal function requires different techniques such as ISCEV Standard pattern ERG or multifocal ERG testing [11, 12].

## Clinical applications of full-field ERGs

Full-field ERG recording enables the distinction between generalized outer and inner retinal dysfunction and between predominantly rod or cone system dysfunction. Symptoms and/or clinical signs may suggest a retinopathy or retinal dystrophy, but the presence, severity and nature of retinal dysfunction cannot always be inferred. ERGs are rarely pathognomonic, but in clinical context can differentiate a wide range of disorders. A list of clinical applications is beyond the scope of this document; typical indications for disorders that are frequently encountered in the electrophysiology clinic and illustrative examples of ERG abnormalities are provided in the ISCEV guide to electrodiagnostic procedures [1].

## Technology

### Electrodes

#### Recording electrodes

Active electrodes connected to the positive input of the recording system may contact the cornea, bulbar conjunctiva or skin on the lower eyelid. These include contact lens electrodes, conductive fibres and foils, conjunctival wire loops and skin electrodes.

Contact lens electrodes provide the highest amplitude; such electrodes should be centrally transparent with an optical opening as large as possible and typically incorporate a device to hold the lids apart. The corneal surface should be protected during use with a non-irritating and non-allergenic ionic conductive solution, e.g. contact lens wetting solutions or artificial tears containing sodium chloride and no more viscous than 0.5% methylcellulose. Topical anesthesia is necessary for contact lens electrodes but may not be required for other types of corneal and conjunctival electrodes. For ERGs recorded with the active skin electrode on the lower eyelid, responses are relatively small compared with corneal recordings and may be less suitable for quantifying abnormalities. Signal averaging may be required to improve the signal-to-noise ratio.

It is incumbent upon practitioners to master the technical requirements of their chosen electrode to obtain good contact, consistent electrode positioning, acceptable electrode impedance and to ensure that waveforms are comparable to those of the ISCEV Standard ERGs.

#### Reference electrodes

Reference electrodes are connected to the negative input of the recording system to enable a potential difference to be recorded. For most types of active electrode, the reference is a skin electrode placed near the ipsilateral orbital rim or outer canthus, temporal to each eye. For recordings made with a contact lens electrode, the reference may be incorporated into the contact lens-speculum assembly in contact with the conjunctiva or may require a separate skin reference electrode.

#### Common electrode

A separate (ground) electrode is attached to an indifferent point and connected to the common input of the recording system. Typical locations are on the forehead, earlobe or mastoid.

#### Skin electrode characteristics

The impedance of passive skin electrodes measured between 10 and 100 Hz should normally be 5 kΩ or less. Ideally, the recording and reference electrodes should have similar impedance levels. The skin should be prepared by cleaning, and a suitable conductive paste or gel (if not integral to the electrode) applied to ensure good electrical connections.

#### Electrode stability

In the absence of light stimulation and eye movement, the baseline voltage should be stable. Reference electrodes may need to be non-polarizable to achieve this stability.

#### Electrode cleaning

Recording ERGs involves the exposure of corneal electrodes to tears, and there is potential exposure of skin electrodes to blood if there is any break in the surface of the skin. Reusable electrodes must be cleaned and sterilized after each use to prevent transmission of infectious agents. The cleaning protocol should follow manufacturers’ recommendations and meet current local and national requirements for devices that contact skin and tears.

### Stimuli

#### Full-field stimulators and light diffusion

Full-field (ganzfeld) stimulation is required and is achieved by using an integrating sphere or dome-like stimulator to provide dispersed light and uniform luminance over the maximal area of retina. A central fixation spot should be provided. Stimulators should allow observation of the patient to monitor fixation and other factors that may influence recordings, e.g. electrode position, eye closure, eye, face and head movements. Ganzfeld stimulators may be large enough to stimulate both eyes simultaneously for two channel recordings, or a small (mini) ganzfeld or optical system (e.g. Maxwellian) can be used for sequential stimulation of each eye. For sequential testing care is needed during stimulation to ensure that light is excluded from the opposite eye to prevent light exposure prior to DA testing.

#### Stimulus duration

Flash duration should be shorter than the integration time of any photoreceptor. The maximum acceptable duration of any stimulus flash is 5 ms.

#### Stimulus wavelength

Flashes and background should be visibly white. Historically, light stimuli specified by ISCEV had broadband spectral content; these stimuli such as xenon flashtubes have largely been superseded by light-emitting diode (LED) technology. LEDs can produce light with a wide range of spectral distributions. Flashes produced by LEDs and by broad-spectrum white flashes that are of equal strength for the cones may be different for the rods, which could result in different ERG reference values. Users should consult manufacturers or make appropriate measurements to establish that the correct corresponding scotopic luminance values are achieved when LEDs are used as a light source. Chromatic (non-white) flashes and backgrounds are not part of the ISCEV Standard ERG, but are used in several ISCEV extended ERG protocols [4–6, 9, 10].

#### Stimulus strength and units

Stimulators should be capable of flashes over a minimum range of 3 log units in strength, in steps of not more than 0.3 log units. The range should ideally be greater, to enable recording of ISCEV extended ERG protocols that involve stronger flashes [7, 8, 10]. Changing background luminance or flash strength should not change the spectral composition.

The background luminance for light adaptation and LA ERG testing is specified in candelas per metre squared (cd·m^-2^). Flash stimuli are quantified by their time-integrated luminance but for simplicity referred to as flash “strength”. This is a measurement of luminous power (flux) per unit solid angle (steradian) per unit surface area, specified in units of candela-seconds per metre squared (cd·s·m^-2^). By convention, the ERG stimulus is named in photopic units even when delivered under dark-adapted conditions. The same flash strength, if expressed in scotopic units for rod-dominated ERGs, is around 2.5 times greater, when using stimuli with appropriate spectral composition. Nominal flash strengths and acceptable ranges are specified in both photopic and scotopic units in Table 1 (see also “Nomenclature” below).

**Table 1:** Stimulus and recording parameters for the ISCEV Standard ERG protocol. Acceptable ranges are defined as ± 10% of nominal values, on a logarithmic scale for flash strengths. For scotopic flash strengths, a scotopic:photopic ratio of 2.5 has been assumed, being typical of xenon flashtubes and therefore providing longitudinal consistency with the extent of rod system stimulation

Type of adaptation; duration of adaptation; abbreviation	Flash strength (acceptable range)		Inter-stimulus interval (frequency)	Recording band pass	
	Photopic (phot cd·s·m^−2^)	Scotopic (scot cd·s·m^−2^)		High pass	Low pass
Dark; ≥ 20 min; DA 0.01 ERG	0.01	0.025	≥ 2 s	≤ 0.3 Hz	≥ 300 Hz
	(0.0063–0.016)	(0.017–0.036)	(≤ 0.5 Hz)		
Dark; ≥ 20 min; DA 3 ERG	3	7.5	≥ 10 s	≤ 0.3 Hz	≥ 300 Hz
	(2.7–3.3)	(6.1–9.2)	(≤ 0.1 Hz)		
Dark; ≥ 20 min; DA 10 ERG	10	25	≥ 20 s	≤ 0.3 Hz	≥ 300 Hz
	(7.9–13)	(18–34)	(≤ 0.05 Hz)		
Dark; ≥ 20 min; DA 3 or 10 OPs	as for DA 3 or 10 ERGs	as for DA 3 or 10 ERGs	as for DA 3 or 10 ERGs	75 Hz	≥ 300 Hz
Light 30 cd·m^−2^; ≥ 10 min; LA 3 ERG	3		≥ 0.5 s	≤ 0.3 Hz	≥ 300 Hz
	(2.7–3.3)		(≤ 2 Hz)		
Light 30 cd·m^−2^; ≥ 10 min; LA 30 Hz ERG	3		30.0 – 36.7 ms	≤ 0.3 Hz	≥ 300 Hz
	(2.7–3.3)		(27 – 33 Hz**)**		

In photometry, the term ‘‘intensity’’ quantifies directional luminous flux per unit area, generally from a point source, and should not be used to describe either the extended, full-field background light or the full-field brief flash stimuli used for ISCEV standard ERG recordings.

#### Nomenclature

Stimulus and response names are described by the state of adaptation and the flash strength in photopic units (phot cd·s·m^-2^) with the understanding that the strength in scotopic units (scot cd·s·m^-2^) will be higher (Table 1). For example, the dark-adapted response to 10 phot cd·s·m^-2^ is called the ‘‘dark-adapted 10 ERG’’ or “DA 10 ERG”. This scheme of naming may also apply to non-standard stimuli, for example those used in extended protocols (e.g. flashes of 30 phot cd·s·m^-2^ used under dark-adapted conditions can be specified as a “DA 30”). Alternatively, non-standard stimuli need not be abbreviated and may be described by specifying the flash strength in phot cd·s·m^-2^. Scotopic units may be used for DA ERGs but this must be explicit, e.g. the dark-adapted 25 scot cd·s·m^-2^ or DA 25 scot cd·s·m^-2^.

By specifying the adaptation state in the current nomenclature, ISCEV specifically avoids naming ERGs as “rod”, “cone”, scotopic or photopic. This reflects the complexity of the waveform generators with different adaptation states. In particular, the light-adapting background suppresses but does not saturate a healthy rod system.

#### Stimulus and background calibration

The user or manufacturer must document the strength of each flash stimulus based on measurements made with an integrating photometer capable of recording the total output of very brief flashes and placed at the location of the eye. The photometer must meet international standards for photometric measurements and have appropriate filters for measurements based on both the photopic and scotopic luminous efficiency functions (luminosity curves). For flashtubes, light output per flash may vary with repetition rate; therefore, separate calibrations may be needed for single flash and for rapidly flickering stimuli. For LED stimulators, output does not vary significantly with repetition rate. The background luminance is measured at the position of the eye with a photometer in non-integrating mode. Users may consult the ISCEV calibration guidelines for details [16]. Responsibility for checking the calibration rests with users. Correct calibration can only be assumed between periods of stable measurements.

### Recording equipment

#### Patient isolation

The patient should be electrically isolated according to current standards for safety of clinical biologic and medical recording systems in the user’s country. In the absence of national requirements, the equipment should meet a general standard such as Medical Electrical Equipment IEC 60601-1.

#### Input characteristics

The recording system should have a minimum input impedance of 10 MΩ (preferably much higher) and be capable of handling the large steady offset voltages that may be introduced by the electrodes. Ideally, the recording system will allow the electrodes to be connected using shielded cables. All amplification and filter characteristics must be matched when two recording channels are used for simultaneously recording ERGs from both eyes.

#### Filtering, frequency bandwidth and sampling

The system should be capable of recording frequencies with a minimum range of 0.3 to 300 Hz and be adjustable. To avoid a loss of information, ERGs should be digitized at a rate of 1 kHz or higher in each channel after appropriate anti-aliasing filtering. The digitizer should have an effective resolution of 1 μV or less.

The recording system should be capable of filtering the ERG signals for such purposes as extracting OPs either prior to recording them or post-acquisition. The band pass for ISCEV Standard ERG recordings, except the OPs, is broad (Table 1). The corner frequencies (3 dB attenuation) are specified as ≤ 0.3 and ≥ 300 Hz. For OPs, the recommended band pass is 75 Hz to ≥ 300 Hz, although filtering between 75 and 100 Hz may be used if characteristics are documented. Roll-off characteristics of the filters are not standardized by ISCEV, but users should be aware that any changes to the filter characteristics or corner frequency of the high- or low-pass filters may change the amplitude and peak times of ERGs. Ideally, comparisons between ERGs should be made with signals filtered in exactly the same way or using post hoc corrections for differences in filter characteristics. Notch (narrow band stop) filters that remove a relatively narrow band of frequencies may improve the appearance of records marred by pickup of mains (general alternating-current power supply; 50 Hz or 60 Hz) or other periodic interfering signals, while distorting the ERG waveform. Such filters should not be used. Post-acquisition methods are more suitable for removing interference of this kind, although it is good practice to minimize interference at source.

#### Real-time display

The recording equipment should automatically provide a visual display so that during testing the operator can continuously monitor the technical quality and stability of ERG recordings, and either coach the patient or make adjustments as necessary to improve recording quality.

#### Recording and storing ERG data

Digital records of all ERGs should be stored. Ideally, these should be records of individual ERG waveforms rather than averages only, which may be distorted by artefact. The duration of each stored record should be at least 300 ms including a pre-stimulus baseline of at least 20 ms to establish stability of the baseline and to help identify stimulus-related artefacts; shorter traces may be displayed in reports. Where feasible, the stimulus time should be indicated for each flash with a marker (for flicker as well as for single-flash ERGs). Each ISCEV standard ERG should be replicated at least once to demonstrate reproducibility, and each replication should be stored.

#### Averaging

Averaging is not normally required to record quantifiable ERGs using corneal or conjunctival ERG electrodes in typical eyes. However, averaging a small number of ERGs reduces variability and background noise and facilitates the estimation of variability. Averaging may be essential to identify and measure pathologic ERGs of low amplitude or ERGs recorded from electrodes on the lower eyelid. Artefact rejection must be a part of any averaging system.

## Clinical protocol

### Preparation of the patient

#### Pupillary dilatation and retinal illumination

Consistent retinal illumination minimizes inter-subject and inter-visit variability. Previous ISCEV standards specified mydriatic pupil dilatation to minimize variability. If mydriasis is used, there is no requirement to adjust stimulus strength according to pupil size, but the pupil diameters should be documented before and at the end of recording and ERGs interpreted accordingly.

Comparable, consistent retinal illumination (constant Trolands) achieved without mydriasis is now accepted but users must ensure that the stimuli and background illumination adequately compensate for the non-dilated pupil with ERG waveforms similar to those recorded with dilated pupils. This requires compensation for pupillary fluctuations and consideration of the Stiles–Crawford effect on cone-mediated ERGs. The reference ranges used should be obtained with the same method and care taken to maintain consistency of methods in monitoring studies. Adjusting flash strength for small pupils measured immediately prior to testing, when mydriasis is not achieved or not possible, may facilitate clinical interpretation, but is not part of this Standard.

#### Pre-exposure to light and other examinations

Indirect ophthalmoscopy, fluorescein angiography, fundus autofluorescence, fundus photography, optical coherence tomography and other imaging techniques that use strong illumination systems should be avoided directly before ERG testing. If these examinations have been performed, a minimum 30 min recovery time is recommended in ordinary room illumination before beginning ERG testing. Scleral depression should also be avoided prior to ERG testing.

#### Adaptation to light or dark

The recordings may start with either LA or DA ERGs, provided the adaptation requirements are met. The ISCEV Standard ERG specifies a minimum of 20 min dark adaptation before recording DA ERGs. If the DA ERGs are recorded first, a minimum of 10 min of light adaptation is specified before recording LA ERGs.

After dark adaptation and for routine applications, the weak flashes should be presented before stronger flash stimuli to minimize the possibility of light adaptation. If contact lens or other corneal electrodes are used, the wearing time can be minimized by dark adapting before inserting the electrodes under dim red light at the end of the adaptation period. Strong red light should be avoided, and a 5 min period of additional dark adaptation is recommended for recovery after insertion of the electrodes.

#### Fixation

Patients should be asked to look at a fixation point. Stable fixation is not as critical as for some other ISCEV standard techniques [11, 12, 15], but eye movements and excessive blinking may have deleterious effects on recordings. These may be due to altering the position of the electrode on the eye, reducing the incident light (e.g. the pupil may be partially obscured by the eyelid if the eyes are elevated) or by causing electrical artefacts. Fixation points should not interfere with dark adaptation (e.g. a weak red LED with a wavelength of 625 nm or longer) and should be visible in light-adapted conditions (e.g. by adjustment to a stronger fixation light or by using a dark fixation spot). Patients who cannot see the fixation point may be asked to look straight ahead and keep their eyes steady. Patients should be monitored to assess compliance and any difficulties in eye opening or fixation should be documented. For non-contact lens electrodes, gentle blinking before each flash may help minimize movement artefacts during the recording.

### The ISCEV Standard ERG protocol

**Fig. 1:**
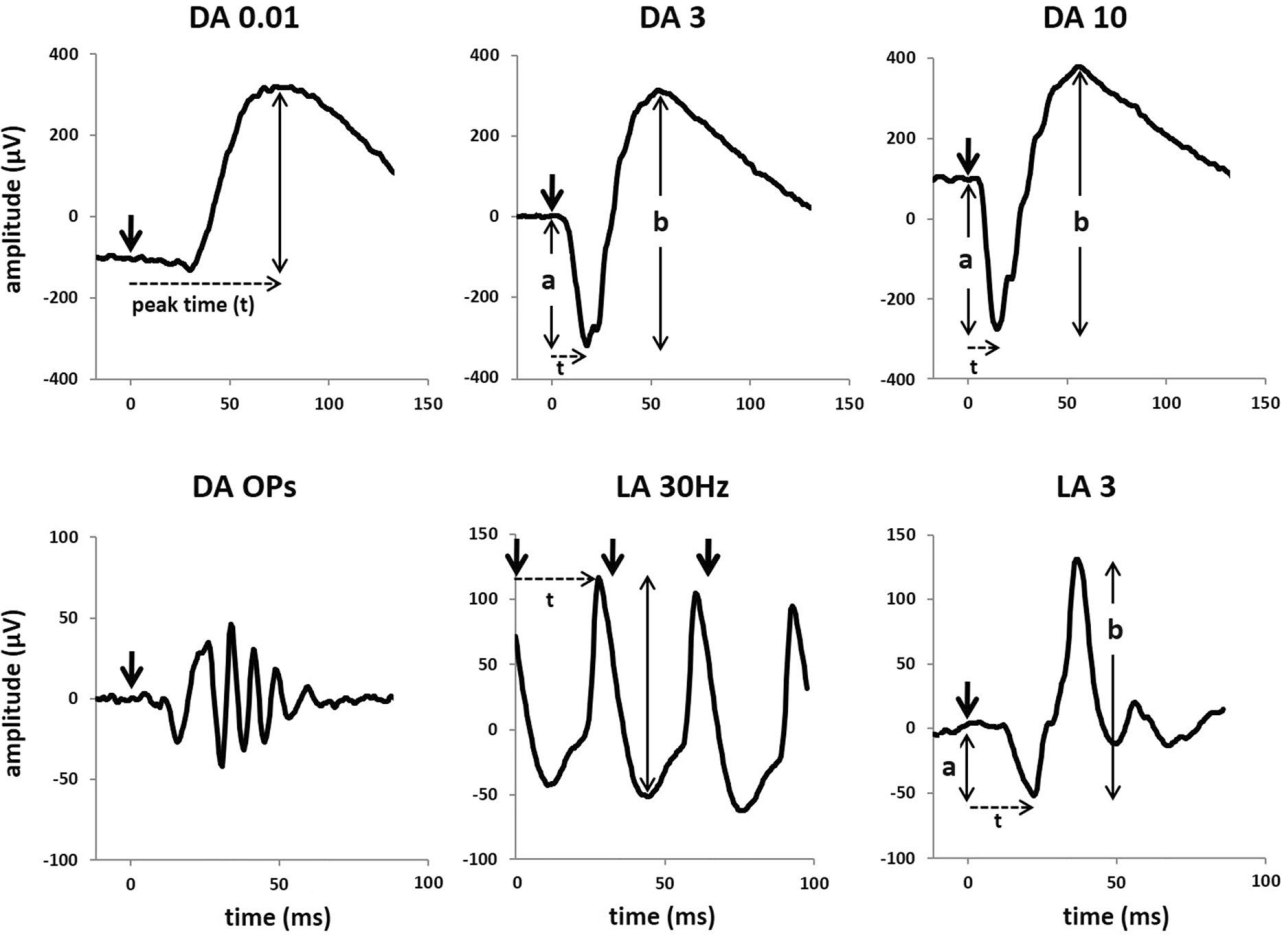
The six ERGs defined by the ISCEV Standard, recorded in this example using a corneal recording electrode. Bold arrows indicate the stimulus flash. The convention is shown for measuring amplitudes (solid vertical lines) and peak times (t; broken horizontal lines) of the standard ERG components, including a-waves and b-waves of dark-adapted (DA) and light-adapted (LA) single flash responses. The amplitude of the LA 30 Hz ERG is measured from trough to peak of a typical wave. The waveforms are examples and do not indicate minimum, maximum or typical values, nor do they show replications as required for reporting

There are six ISCEV Standard ERGs (Fig. 1 and Table 1), including five named according to the state of dark- or light- adaptation and flash strength delivered; the flicker ERG is named according to the adaptation state and stimulus frequency (in Hz).

#### Dark-adapted 0.01 ERG (DA 0.01 ERG)

After a minimum of 20 min dark adaptation, the DA 0.01 ERG is recorded before ERGs to stronger flashes, since it is the most affected by light adaptation. The stimulus is a relatively weak white flash of 0.01 phot cd·s·m^-2^ with a scotopic strength near 0.025 scotopic cd·s·m^-2^ (Table 1). The minimum interval between flashes is 2 s.

#### Dark-adapted 3 ERG (DA 3 ERG)

The DA 3 ERG is typically recorded after the DA 0.01 ERG since light adaptation by the weaker stimulus is not clinically significant. The strength of the flash is 3 phot cd·s·m^-2^ (near 7.5 scotopic cd·s·m^-2^) (Table 1). The minimum interval between successive stimulus flashes is 10 s.

#### Dark-adapted 10 ERG (DA 10 ERG)

The DA 10 ERG is typically recorded after the DA 3 ERG. The flash strength is 10 phot cd·s·m^-2^ (near 25 scotopic cd·s·m^-2^)  (Table 1). The minimum interval between successive flashes is 20 s.

#### Dark-adapted oscillatory potentials (DA OPs)

The DA 3 and DA 10 OPs are recorded to the 3 or 10 cd·s·m^-2^ flash stimuli and may be obtained as separate recordings or derived from ERG waveforms by filtering, post-acquisition. Either DA 3 OPs or DA10 OPs may be extracted and used, providing the stimulus strength is specified, that quantified values are compared with appropriate reference data and that serial studies consider consistency of methods. The characteristics of the OPs change after the first stimulus and amplitudes increase with longer inter-stimulus intervals. Only the OPs to the second and subsequent flashes should be retained or averaged.

#### Light-adapted 3 ERG (LA 3 ERG)

The LA 3 ERG may be recorded before or after the LA 30 Hz ERG. If recorded after the DA ERGs, a minimum of 10 min light adaptation with a full-field standard light-adapting background of 30 cd.m^-2^ is required before recording LA ERGs. If recorded before DA ERGs in a patient adapted to typical room light, one minute of exposure to the standard background is sufficient. The LA 3 ERG is a single flash response to a 3 cd·s·m^-2^ flash superimposed on the constant standard background (30 cd·m^-2^). The minimum interval between successive flashes is 0.5 s.

#### Light-adapted 30 Hz ERG (LA 30 Hz ERG)

LA 30 Hz flicker ERGs are obtained under the same conditions of light adaptation as the LA 3 ERG using a train of brief (< 5 ms), full-field flashes of 3 phot cd·s·m^-2^ superimposed on the standard background (30 cd·m^-2^). The flash rate is approximately 30 stimuli per second (acceptable range 27–33 Hz). Laboratories should choose a consistent frequency, avoiding exact multiples of the line frequency of the electricity supply or of local instruments. The initial onset of a flickering stimulus includes a transient response from the light-adapted rods and these initial responses are excluded so that stable conditions are reached. Laboratories may incorporate pauses of ≥ 300 ms between short bursts of flicker so that this initial waveform can be identified.

### Analysis and reporting

#### Single flash ERGs

The conventions for measurement of the ISCEV ERG peak times (also called implicit times) and amplitudes are illustrated in Fig. 1. The b-wave of the DA 0.01 ERG is relatively broad in shape and is measured to the peak (or midpoint) of the response. For the DA 3, DA 10 and LA 3 ERGs, the a-wave amplitude is measured from the average, pre-stimulus baseline to the a-wave trough; the b-wave amplitude is measured from the a-wave trough. The a- and b- wave peak times are measured from the flash to the trough or peak of the wave. Peak times will depend on flash duration if measured from the beginning or end of the stimulus flash. This effect is sufficiently small when the stimulus duration is less than a millisecond and can be ignored, for example, when stimuli are generated by a xenon flashtube. For longer flashes of up to 5 ms, such as those generated by LEDs, the time to peak should be calculated from the midpoint of the flash. However, it is highlighted that the slope of the leading edge of the a-wave varies with stimulus duration.

The DA 3 ERG a-wave has a double trough in a high proportion of healthy individuals. Either or both can be measured but the measured components should be specified. Abnormal DA and LA ERGs may also have abnormally complex or polyphasic waveforms that are not easily characterized in simple terms, e.g. by reference to a- and b- waves or a single trough or peak; a qualitative description can be given and the components or sub-components that are quantified specified.

#### DA OPs

There are typically three main positive peaks, often followed by a fourth peak that is smaller. For routine applications, qualitative analysis is acceptable and the presence and waveform of the OP peaks and their normality relative to appropriate reference data may be adequate for most clinical applications. Quantification is optional, but if used must specify the filter characteristics and measurement methods, e.g. individual peak amplitudes measured from their preceding troughs, sum of amplitudes of specified peaks or integrated root-mean-square amplitude of the OP waveforms.

#### Flicker ERGs

The amplitude of a flicker ERG is measured from trough to peak of a typical wave (Fig. 1). Care should be taken to avoid the largest or smallest peak-to-trough amplitude. The response to the initial onset of the flicker, which may resemble a single flash ERG, should always be excluded. The peak time of the flicker ERG is measured from the midpoint of the stimulus flash to the following peak (avoiding the initial waveform). It is helpful to average several typical measurements to determine the peak timing and amplitude of the flicker ERG. Abnormality of waveform shape should be described (e.g. a double-peak waveform), and the components that are measured clearly identified.

An alternative to conventional flicker ERG peak time and amplitude measurement is to use frequency domain analysis, which provides the magnitude and phase at the stimulation frequency and optionally for higher harmonic components of the waveform. The magnitude and phase of an individual harmonic component does not relate directly to amplitude and peak times of the flicker ERG in the time domain, unless all substantial harmonic components are included.

### Reference values

Reference ranges (previously termed ‘normative’ data) for standard ERGs are specific to the type of electrode, and each laboratory should use suitable reference data to interpret patient ERGs. Establishing reference values involves recruiting and testing sufficient reference subjects per clinically relevant partition (e.g. sex). Subjects should be matched to the patient population in demographic factors. Reference limits should be constructed to enclose the central 95% of values, i.e. the 2.5th and 97.5th percentile. Nonparametric or robust techniques are likely to be more appropriate than parametric techniques. ERG parameters mature rapidly during infancy and there are age-associated changes throughout life: robust curve fitting may be a useful aid to interpretation, avoiding partitioning of a continuous variable.

Establishing laboratory-specific reference values is the optimal process. If external reference data are used, for example, manufacturers’ data or published data, they must be verified as appropriate for local use with an understanding of possible limitations and how reference limits were defined.

Legacy reference data may be transferred and validated, for example in response to a change of instruments, but the methods should be stated. In longitudinal studies, subject-specific reference values are typically more sensitive to change than comparisons with cross-sectional reference values for the population. Such assessments should consider the consistency of stimuli and recording methods, and inter-session variability due to electrode position, pupil diameter, age, etc. Inter-ocular comparisons may also be of value if dysfunction is asymmetrical or unilateral.

### Reporting for the ISCEV Standard ERG protocol

The current ISCEV Standard includes some options for stimulus and recording parameters and it is essential that the methods adopted are described and recordings compared with appropriate reference data. Stimuli are specified according to the standard nomenclature, e.g. DA 0.01, DA 10, etc. If ERGs were recorded without mydriasis the method of achieving equivalent stimuli must be stated. Interpretation of abnormal data should consider the effects of pupil size and any limitations of the method used.

ISCEV Standard ERG reports should display representative waveforms of each of the patient’s standard ERGs with amplitude and time calibrations, list the stimulus variables and report the state of light- or dark adaptation. A minimum of two responses from each stimulus condition should be displayed to demonstrate the degree of consistency. ERG waveforms should include at least 20 ms of baseline prior to the stimulus for single flash ERGs and, where feasible, should indicate the stimulus time for each flash with a marker (for flicker as well as for single flash ERGs). The time-integrated luminance values (in phot cd·s·m^-2^) of the stimulus flashes and the background luminance (phot cd·m^-2^) should be reported with the option to also specify the scotopic units (scot cd·s·m^-2^) for the DA ERGs. Reports should indicate explicitly whether there were any deviations from the current ISCEV Standard in techniques or recording parameters. All reports should list results along with reference values and ranges. Provenance of reference values should be stated briefly. Reports should note the time of testing, pupil diameters and the type and position of the active electrode. Conditions not specified by the Standard such as sedation or anesthesia, and the level of compliance should also be documented.

## Pediatric and other non-standard ERG recording protocols

### Pediatric ERG recording

ERGs can be recorded from infants and young children, but interpretation of results must take into account any variations in recording methods, compliance and age-appropriate reference data.

ERGs mature during infancy, and signals from very young infants must be interpreted with caution. Later in infancy and childhood, ERGs approach adult waveforms and amplitudes. Specifically, somewhat lower ERG amplitudes and longer peak times generally apply below 6–12 months of age under dark-adapted conditions, and below 2–3 months of age under light-adapted conditions. Before 6 months of age, the DA 0.01 ERG may be poorly defined in healthy infants; DA 3 and DA 10 ERGs are usually well defined at all ages in infants without retinal disease.

Most pediatric patients can be studied without sedation or general anesthesia. Small infants can be swaddled if needed. Non-compliant patients may be tested with oral sedation or general anesthesia; appropriate medical supervision and local guidelines must be followed with respect to indications, risks, medical monitoring requirements and the choice of a sedative/relaxant versus general anesthesia. Interpretation should take into account the possible effects of sedation or anesthesia on ERG waveforms.

If contact lens electrodes are used, pediatric sizes will be required for infants and young children. Other types of corneal and skin electrodes vary in their applicability to children; greater comfort may be offset by greater electrode movement or smaller ERG amplitudes. To minimize artefact, special care is required with children to monitor electrode position and compliance. Limited compliance can make pediatric records variable, and several repetitions of each ERG should be recorded to recognize reproducible waveforms. Shortened protocols may be appropriate to obtain the ERGs most critical to the diagnostic question under investigation, e.g. see suggested abbreviated protocol below. Reports should note the methods used, degree of cooperation and any relevant medications.

### Non-standard abbreviated ERG protocol

The ISCEV Standard ERG protocol may not be possible for all patients, for example due to limited ability to comply with testing or in young children. The abbreviated protocol suggested below is intended for use only when the ISCEV Standard method is precluded, with a view to promote convergence of short ERG protocol alternatives.

This abbreviated protocol includes the use of Ganzfeld stimulation, but unlike the ISCEV Standard protocol, dark adaptation is reduced to 10 min, fewer DA ERGs are recorded and mydriasis is not required.

The LA ERG is recorded first to obviate the need for a specific period of light adaptation. It is recognized that a shorter period of dark adaptation may be all that is possible or practical in some patients, and a qualitative analysis may be sufficient to support some diagnoses and to inform clinical management in some cases. All other requirements are identical to those of the ISCEV Standard ERG protocol, including the electrode requirements and the stimulus and recording parameters, but with natural or dilated pupils. Four conditions specified below are suggested in the order given:

- LA 3 ERG
- LA 30 Hz ERG
- DA 0.01 ERG following 10 min dark adaptation
- DA 10 ERG

### Reporting of the non-standard abbreviated ERG protocol

Reporting should follow that for the ISCEV Standard ERG. It is essential that the use of an abbreviated protocol, the dark adaptation time and state of the pupil (natural or dilated with diameter) are explicitly stated. Reference ranges should be obtained using the same methods, or may be adapted from those for the ISCEV Standard ERG protocol, if validated. It is recognized that ERGs will be influenced by a lack of mydriasis and that for a dark adaptation period of 10 min, DA 0.01 ERGs are likely to be attenuated compared with ERG waveforms obtained after longer dark adaptation. However, reducing the dark adaptation period from 20 to 10 min is unlikely to affect the DA 10 ERGs in healthy individuals. Interpretation of abnormal data should take into account the shortened period in the dark, e.g. in disorders that impair the dark adaptation of photoreceptors.

## Abbreviations

ERG: Electroretinogram
ISCEV: International Society for Clinical Electrophysiology of Vision
DA: Dark-adapted
LA: Light-adapted
OP: Oscillatory potential
VEP: Visual evoked potential
LED: Light-emitting diode

## Declarations

### Statement on the welfare of animals

This article does not contain any research studies with animals performed by any of the authors.
